# Hedgehog Pathway Inhibition Hampers Sphere and Holoclone Formation in Rhabdomyosarcoma

**DOI:** 10.1155/2017/7507380

**Published:** 2017-01-24

**Authors:** Ana Almazán-Moga, Patricia Zarzosa, Isaac Vidal, Carla Molist, Irina Giralt, Natalia Navarro, Aroa Soriano, Miguel F. Segura, Arantza Alfranca, Javier Garcia-Castro, José Sánchez de Toledo, Josep Roma, Soledad Gallego

**Affiliations:** ^1^Laboratory of Translational Research in Child and Adolescent Cancer, Vall d'Hebron Research Institute, Hospital Universitari Vall d'Hebron, Universitat Autònoma de Barcelona, Barcelona, Spain; ^2^Cellular Biotechnology Unit, Instituto de Salud Carlos III, Madrid, Spain; ^3^Pediatric Oncology and Hematology Department, Hospital Universitari Vall d'Hebron, Universitat Autònoma de Barcelona, Barcelona, Spain

## Abstract

Rhabdomyosarcoma (RMS) is the most common type of soft tissue sarcoma in children and can be divided into two main subtypes: embryonal (eRMS) and alveolar (aRMS). Among the cellular heterogeneity of tumors, the existence of a small fraction of cells called cancer stem cells (CSC), thought to be responsible for the onset and propagation of cancer, has been demonstrated in some neoplasia. Although the existence of CSC has been reported for eRMS, their existence in aRMS, the most malignant subtype, has not been demonstrated to date. Given the lack of suitable markers to identify this subpopulation in aRMS, we used cancer stem cell-enriched supracellular structures (spheres and holoclones) to study this subpopulation. This strategy allowed us to demonstrate the capacity of both aRMS and eRMS cells to form these structures and retain self-renewal capacity. Furthermore, cells contained in spheres and holoclones showed significant Hedgehog pathway induction, the inhibition of which (pharmacologic or genetic) impairs the formation of both holoclones and spheres. Our findings point to a crucial role of this pathway in the maintenance of these structures and suggest that Hedgehog pathway targeting in CSC may have great potential in preventing local relapses and metastases.

## 1. Introduction

Rhabdomyosarcoma (RMS) is the most common type of soft tissue sarcoma in children and is considered one of the most prevalent pediatric extracranial solid tumors. RMS can be divided into two main histopathologic subtypes: embryonal and alveolar (eRMS and aRMS, resp.). These subtypes differ considerably in their clinical phenotype and molecular features. From a molecular point of view, the majority of aRMS (80% to 85%) contain one of the reciprocal chromosomal translocations: either t(2;13)(q35;q14) or t(1;13)(p36;q14). These translocations generate the anomalous fusion genes* PAX3-FOXO1* and* PAX7-FOXO1*, respectively [1, 2]. However, no characteristic translocations have been described in eRMS. The embryonal subtype is characterized by the loss of heterozygosity in the short arm of chromosome 11 (11p15.5) [3] and gains in chromosomes 2, 7, 8, 11, 12, 13, and 17 [4].

All the cells contained in a tumor are not identical and exhibit diverse proliferative and differentiation potential, a feature commonly referred to as tumor heterogeneity. The existence of a small fraction of cells—called cancer stem cells (CSC)—with hierarchical organization and responsible for the onset and propagation of cancer was first demonstrated in acute myeloid leukemia [5]. Later, the existence of cancer stem cells was also described in solid tumors such as prostate cancer [6], melanoma [7], brain tumors [8] and breast cancer [9]. According to the clonal evolution model, some tumor cells acquire sequential mutations which progressively confer on them a growth advantage, thereby promoting tumor progression [10, 11]. On the other hand, the cancer stem cell hypothesis suggests the existence of a rare subpopulation of cells with properties of normal stem cells—self-renewal, high differentiation potential, proliferation, and chemoresistance—able to initiate a tumor and produce cellular heterogeneity. Therefore, this model points to cancer stem cells as the only cell type with actual tumorigenic potential. Moreover, this subpopulation could also be, always according to this theory, responsible for the formation of local relapses and metastases [10–12]. However, these two models are not mutually exclusive, since tumor stem cells may also undergo clonal evolution.

Childhood cancers have specific characteristics that define them as entities differing strongly from adult malignancies owing to their different etiology, biology, response to treatment, and outcome. Given these differences, we must question whether cancer stem cells exist in pediatric cancers and, if so, whether their function is similar in pediatric and adult malignancies. For the particular case of RMS, controversy exists as to suitable markers for stem cell isolation. Thus, Hirotsu et al. [13] were the first to describe the existence of tumor-initiating cells in eRMS and proposed FGFR3 as a suitable marker for their isolation. Later, Walter et al. rebutted the possibility of using FGFR3 as a stem cell marker in RMS, and proposed CD133/2 as an isolation marker for this subpopulation [12]. Pressey et al. [14] confirmed the presence of CD133-positive myogenic precursors in RMS cell lines. However, a recent work demonstrated that CD133-positive cells were not able to generate tumors more efficiently (than CD133 negative cells) in immunodeficient mice. Those authors proposed the transcription factor SOX2 as a cancer stem cell marker in RMS, Ewing's sarcoma and osteosarcoma [15]. Interestingly, the existence of CSC in alveolar RMS has not been demonstrated to date despite the efforts of the scientific community working in this field, indicating the possibility of a lack of suitable markers to identify this rare subpopulation.

The method of stem cell enrichment based on the ability of neural stem cells to grow in suspension, forming characteristic spheroids, was first described by Reynolds and Weiss [16]. It is considered that only stem cells have the ability to form spheres when seeded at low density [17]. In recent years, this property has been confirmed in different malignancies of the nervous system and breast cancer, among others [18, 19]. Recently, the existence of spheres enriched in tumor-initiating cells and overexpressing the epitope CD133/2 has been reported in eRMS [12]. An alternative test is the colony-formation capacity of these cells, which depends on the proliferation and differentiation potential of selected cells [11]. The classic test involves seeding cells at low density, a condition under which stem cells have greater ability to form colonies. Furthermore, a relationship between the shape of the colonies and their stem cell potential has also been described, thus revealing the existence of three types of clones with very different proliferative capacity [20]. According to the compaction, contour, and proliferation capacity of the colonies, those authors then classified the three types into holoclones, meroclones, and paraclones. The holoclones (small and compact, forming a well-defined boundary) had great ability to replicate. Conversely, paraclones were formed by scattered cells in clones with indefinite borders which showed very low proliferative capacity. The third type of colony containing a mixture of cells with different proliferative capabilities was a transition between holoclones and paraclones. Some years later, another study revealed that holoclones generated from keratinocytes were enriched in stem cells and conserved their capability to self-renew. Moreover, only stem cells from the follicle were able to form holoclones [21]. In terms of cancer, several studies have shown that holoclones formed by glioma cells, prostate cancer and pancreatic cancer are the only ones able to be tumorigenic [22–24]. The ability of cells to form holoclones in pediatric sarcomas has not been reported to date.

Some signaling pathways, characterized by their capacity to orchestrate normal stem cell self-renewal and proliferation, are also crucial to initiate tumors when they are deregulated. Among them, the Hedgehog (HH) pathway has been shown to be preponderant in stem cell self-renewal programs [11]. Recently, involvement of this pathway in stem cell maintenance in various cancers has also been demonstrated [25]. HH signaling was first described in* Drosophila *[26] and* HH *genes are considered to be key regulators of several development processes.* HH* signaling also plays important roles in adult organisms such as stem cell maintenance, tissue repair and regeneration. In the absence of active* SMO* in the membrane,* GLI* family* (GLI1, GLI2*,* and GLI3)* are proteosomically processed. Upon binding of an* HH* ligand, active* SMO* is detected in the membrane and prevents* GLI *proteosomal processing.* GLI* is then translocated to the nucleus where it binds to* GLI*-specific promoters [27–29]. The initial link between* HH* signaling and human cancers was established when mutations in human* PTCH1* were found to be associated with a rare hereditary disease called Gorlin's syndrome. Patients with Gorlin's syndrome have a high incidence of basal cell carcinoma, medulloblastoma and RMS [30–32]. However, mutation of components of the HH pathway in RMS is rare, except in Gorlin's syndrome that accounts for a very low percentage of RMS patients. HH pathway alterations, typified by loss of function of PTCH and SUFU or activating mutations in* SMO*,* HH*, or* GLI*, are thought to be oncogenic in a considerable number of other cancers [33]. Currently, consistent activation of the pathway is well established and generally accepted in RMS [34]. Moreover, several publications combining in vitro and in vivo works with xenografted RMS models agreed on the possibility of effectively reducing tumor growth by* HH* pathway inhibition [35–37]. During myogenesis, the* HH* pathway is involved in regulating the preservation, expansion, and differentiation of skeletal muscle progenitor cells. The fact that RMS presents an aberrant regulation of this signaling pathway suggests it may play a role in the origin of this sarcoma and in the maintenance and renovation of this rare cell subpopulation [38]. Satheesha et al. [39] showed that* HH* signaling modulates eRMS tumor-initiating cell self-renewal. However, no previous works studied activation of the pathway in aRMS spheres or holoclones in either RMS subtype.

The main aim of this work was to demonstrate the presence of specific subpopulations of cells with CSC characteristics in RMS by describing the capability of RMS cells to generate holoclones and spheres as well as the prominent* HH* pathway activation in cells contained in these supracellular structures. Both aRMS and eRMS were found to have different cell populations able to form holoclones, paraclones, and meroclones as well as spheres. The role of the* HH* pathway was characterized in these particular subpopulations, which pointed to a crucial role of this pathway in their maintenance.

## 2. Materials and Methods

### 2.1. Cell Culture

RH30 (aRMS, PAX3/FOXO1 translocation), RD (eRMS), and HTB82 (eRMS) cell lines were obtained from American Type Culture Collection (ATCC) and CW9019 (aRMS, PAX7/FOXO1 translocation) was generated in Dr. Jaclyn Biegel's laboratory. All RMS cell lines were grown in MEM media (Biowest), supplemented with 10% fetal bovine serum (Sigma-Aldrich), 2 mM L-glutamine, 1 mM sodium pyruvate, 1x nonessential amino acids, 100 U/mL penicillin, and 0.1 mg/mL streptomycin (all reagents from Biowest). Cells were maintained at 37°C in a 5% CO_2_ water-jacketed incubator.

### 2.2. Drug Treatments

The* SMO* inhibitor Sonidegib (LDE225) was purchased from Selleckchem. The* SHH* and* IHH* blocking antibody MEDI-5304 was kindly provided by MedImmune. Working concentrations were 15 *μ*M for Sonidegib and 30 *μ*g/mL for MEDI-5304. The control plates were treated with equal volumes of vehicle. Cells were pretreated for 48 h before all functional assays. Cells were not treated during sphere- and holoclone-formation assays.

### 2.3. Analysis of Cell Viability

Cells were incubated with 2 *μ*g/mL propidium iodide and analyzed by flow cytometry in a FACSAria cytometer (BD Bioscience). Results were analyzed with FCS Express 4 Flow Cytometry software (De Novo Software).

### 2.4. Plasmids, Lentiviral Production, and Transduction

Genetic inhibition of the* HH* ligands and* GLI1* was performed by shRNAs cloned into the lentiviral vector pGIPZ (GE Dharmacon). The empty vector was used as a control. Briefly, the cell line used to produce the lentiviral particles (HEK 293T) was transfected with the envelope plasmid pMD2G (4 *μ*g), the packaging plasmid psPAX2 (8 *μ*g), and pGIPZ transfer vector (12 *μ*g), according to the Lipofectamine 2000 Transfection Reagent manufacturer's instructions (Thermo Fisher Scientific). Cell media containing viral particles were removed after 24 h. After 48 h of infection, positively transduced cells were selected with puromycin (1 *μ*g/mL, Sigma-Aldrich). Knockdown efficiency of each shRNA was analyzed by western blot. The GE Dharmacon IDs of selected clones were V3LHS_82400 (shSHH), V3LHS_336297 (shIHH), V3LHS_401021 (shDHH), and V2LHS_262249 (shGLI1). Functional assays were performed 7 days after infection.

### 2.5. Clonal Selection

Viable cells were sorted and seeded at clonal density with FACSAria flow cytometry (BD Bioscience) in 96-well plates. All apoptotic or nonviable cells were discarded. After 10 days of incubation, clones were classified into holoclones, meroclones, or paraclones according to phenotypic criteria. For secondary colony formation, clones were trypsinized and seeded again at clonal density in 96-well plates. Total number and typology of the clones obtained were assessed in triplicate for each cell line. For RNA extraction, clones were trypsinized and expanded in 6-well plates.

### 2.6. Sphere-Formation Assay

10^4^ CW9019 and 10^3^ HTB82 cells were grown in neurobasal media (Thermo Fisher Scientific) supplemented with 2x B27 (Gibco), 2 mM L-glutamine, 100 U/mL penicillin, 0.1 mg/mL streptomycin (Biowest), 20 ng/mL Epidermal Growth Factor (EGF, R&D System), and 10 ng/mL Fibroblast Growth Factor (FGF, Sigma) in 6-well plates and maintained at 37°C in a 5% CO_2_ water-jacketed incubator for 1 week.

For secondary sphere formation, spheres were collected, mechanically disaggregated, and seeded at the above-mentioned density. The number of secondary spheres formed was counted after 1 week of incubation. The sphere-formation efficiency of each cell line was tested in triplicate.

### 2.7. RNA Extraction, Retrotranscription, and Real-Time PCR

Total RNA from cell lines was extracted using the RNeasy Mini Kit (Qiagen), following the manufacturer's instructions. 2 *μ*g of total RNA was incubated with random primers (Invitrogen) for 5 min at 70°C and reverse-transcripted using 200 U of Moloney murine leukemia virus reverse transcriptase (Promega) for 1 h at 37°C. A 40-cycle PCR based on the TaqMan assay (Applied Biosystems) was performed to detect* SHH, IHH, DHH*, and* GLI1* (Hs00179843_m1, Hs00745531_s1, Hs00368306_m1, and Hs00171790_m1 TaqMan assays, resp.). The housekeeping gene* TBP* (assay Hs00172424_m1) was used as internal control. Relative levels of each mRNA analyzed were quantified by the 2^−ΔΔCt^ method of Livak and Schmittgen [40]. All samples were tested in triplicate.

### 2.8. Statistical Analyses

Statistical analyses were performed using GraphPad Prism software. All data were presented as mean ± SEM. Statistical significance was determined by Student's *t*-test. *p* values were considered significant at ^*∗*^*p* < 0.05; ^*∗∗*^*p* < 0.01; ^*∗∗∗*^*p* < 0.001.

## 3. Results

### 3.1. RMS Cells Are Able to Form Holoclones and Spheres

**Figure 1:**
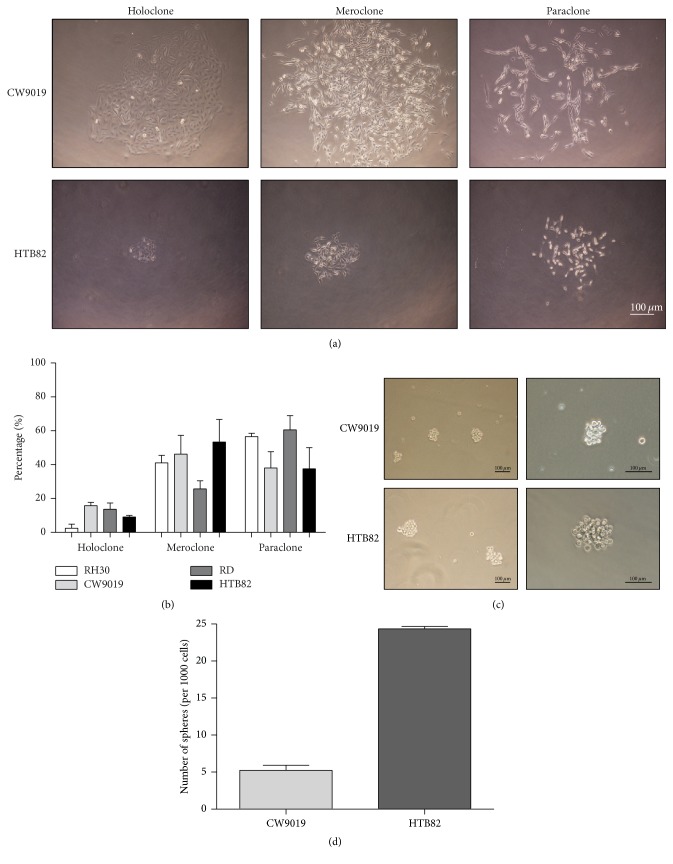
Formation of holoclones and spheres in RMS cell lines. (a) Representative images of holoclones, meroclones, and paraclones in CW9019 and HTB82 cell lines. (b) Percentages of clones classified as holoclones, meroclones, or paraclones in CW9019, HTB82, RD, and RH30 cell lines. (c) Representative images of CW9019 and HTB82 spheres. (d) Number of spheres formed per 1000 cells seeded in neurosphere media. Data were expressed as mean ± SEM of three independent experiments.

RMS cell lines CW9019, HTB82, RD, and RH30 were seeded at clonal density to assess their heterogeneity in colony formation. After approximately 10 days of incubation, all cell lines showed three distinct colony types based on morphologic criteria. Compact colonies with clear contours corresponded to holoclones, whereas colonies formed with separated cells corresponded to paraclones; intermediate colonies were meroclones (Figure 1(a)). While meroclones and paraclones were quite frequent (25–60%), holoclones were the less frequent colony type (1–16%) (Figure 1(b)). Interestingly, paraclones were not able to grow indefinitely, and never formed colonies with more than 40–50 cells, approximately. Given their capacity to clearly form the three types of clones, CW9019 and HTB82 were selected for subsequent studies. Both cell lines were also able to form spheres (Figure 1(c)). The cell line HTB82 showed sphere-formation efficiency 5-fold greater than CW9019 cells (25 spheres/1000 cells versus 5 spheres/1000 cells) (Figure 1(d)).

### 3.2. RMS Holoclones and Spheres Were Able to Self-Renew

**Figure 2:**
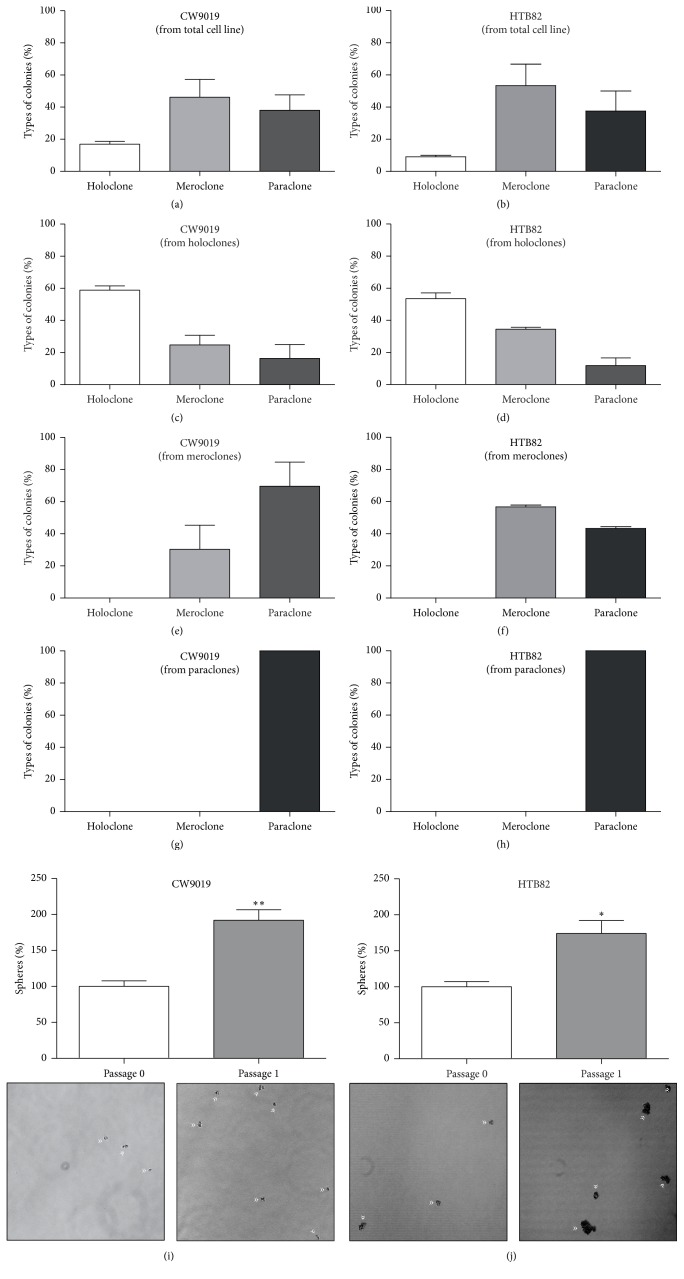
Self-renewal capacity of cells contained in RMS holoclones and spheres. ((a) and (b)) Initial percentage of the three types of clones generated in CW9019 and HTB82 cell lines, respectively. Representative images and quantification of secondary clones formed from holoclones (c and d), meroclones (e and f), and paraclones (g and h) in CW9019 and HTB82 cell lines, respectively. Enrichment in the secondary sphere fraction obtained in CW9019 (i) and HTB82 (j) cell lines. All experiments were tested in triplicate. Significance: ^*∗*^*p* < 0.05; ^*∗∗*^*p* < 0.01.

Self-renewal is one of the hallmarks of stem cells. Confirming that cells contained in holoclones and spheres have self-renewal potential is the clue to assessing their stem cell potential. The three types of clones were detached, disaggregated, and reseeded at clonal density and secondary clones were classified and counted. In the two cell lines analyzed, paraclone-derived cells exclusively produced paraclones (Figures 2(g) and 2(h)), meroclone-derived cells formed both meroclones (30.3% and 56.7%, resp.) and paraclones (69.7% and 43.3%, resp.) (Figures 2(e) and 2(f)), whereas holoclone-derived cells were able to generate the three types of colonies: 59–53.6% holoclones, 24.8–34.5% meroclones, and 16.2–11.9% paraclones, in the cell lines CW9019 and HTB82, respectively (Figures 2(c) and 2(d)). Interestingly, the typology of colonies formed from a holoclone presented marked enrichment in holoclones (from 16% to 59% in CW9019 and from 9% to 53.6% in HTB82) compared to the initial percentages (Figures 2(a) and 2(b)). Similarly, after disaggregation and reseeding of spheres, a significant increase in sphere number was observed in the two cell lines analyzed (Figures 2(i) and 2(j)).

### 3.3. The Main Effector of* HH* Pathway* (GLI1)* Is Upregulated in RMS Holoclones and Spheres

**Figure 3:**
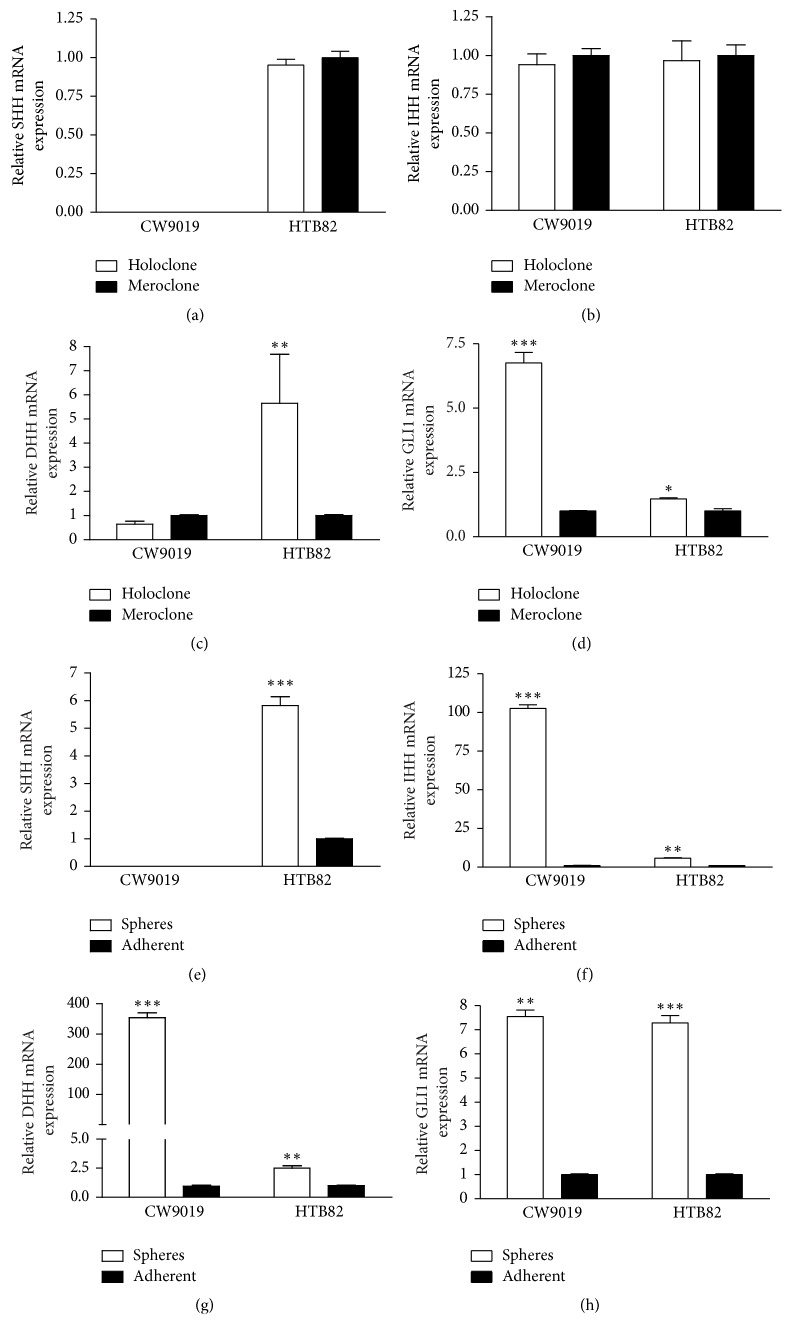
RMS stem-like cells showed* GLI1* upregulation. Analysis of SHH, IHH, DHH, and* GLI1* mRNA levels in holoclones (a, b, c, and d) and spheres (e, f, g, and h) of CW9010 and HTB82 cell lines. Values were referred to expression levels of meroclones and adherent cells (black bars), respectively. Data were tested in triplicate and expressed as mean ± SEM. Significance: ^*∗*^*p* < 0.05; ^*∗∗*^*p* < 0.01; ^*∗∗∗*^*p* < 0.001.

The mRNA levels of* HH* ligands—*SHH, IHH*, and* DHH*—and the* HH* target gene* GLI1* were analyzed in CW9019 and HTB82 holoclones and spheres and compared to the levels in meroclones or adherent cells, respectively. Although holoclones presented high expression levels of* HH* ligands (results not shown), their levels were similar to those of meroclones, with the exception of* DHH*, which showed clear upregulation in HTB82 holoclones (Figures 3(a), 3(b), and 3(c)). Spheres showed significant upregulation of the three HH ligands in both cell lines (Figures 3(e), 3(f), and 3(g)). However,* GLI1* was clearly upregulated in both holoclones and spheres (Figures 3(d) and 3(h)), suggesting that* HH* pathway activation may play an important role in stemness maintenance in RMS cells.

### 3.4. *HH* Pathway Inhibition Impaired the Formation of RMS Holoclones and Spheres

**Figure 4:**
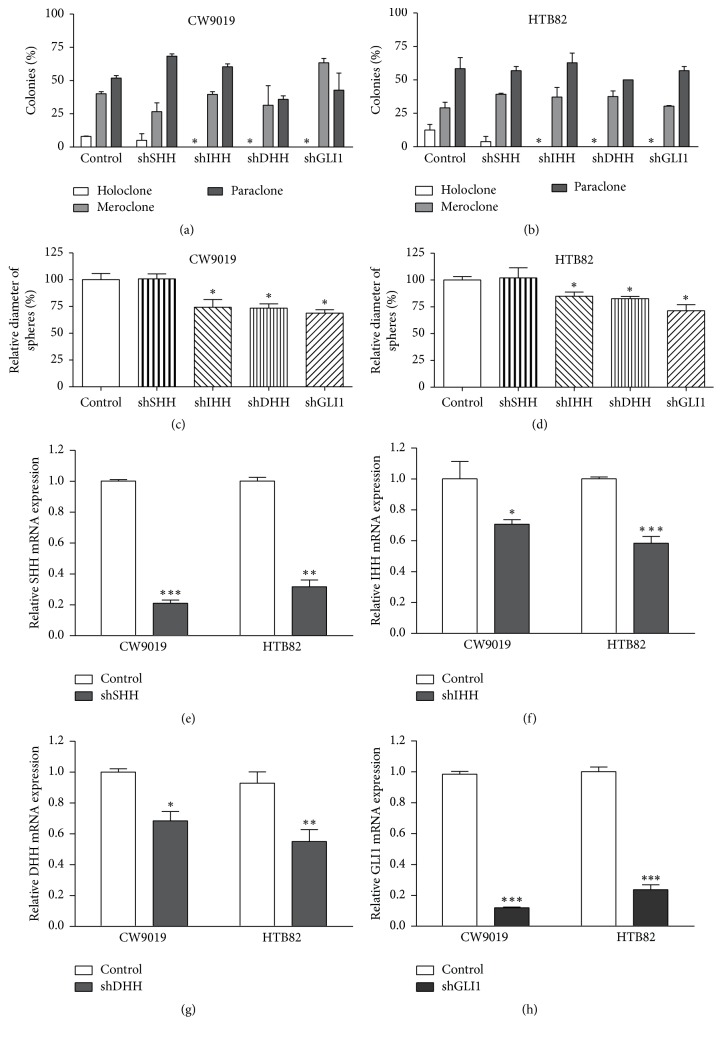
Effects of HH ligands and* GLI1* depletion on RMS holoclone and sphere formation. ((a) and (b)) Plots representing the percentage of each colony type formed by* SHH*,* IHH*,* DHH*, and* GLI1* shRNA-expressing CW9010 and HTB82 cells, respectively. ((c) and (d)) Plots representing the relative diameter of spheres formed by shRNA-expressing cells. Values were referred to control cells (transfected with pGIPZ empty vector) and expressed as mean ± SEM of three independent experiments. From (e) to (h) real-time PCRs to assess the downregulation of all shRNA targets:* SHH, IHH, DHH*, and* GLI1*, respectively. Significance: ^*∗*^*p* < 0.05; ^*∗∗*^*p* < 0.01; ^*∗∗∗*^*p* < 0.001.

**Figure 5:**
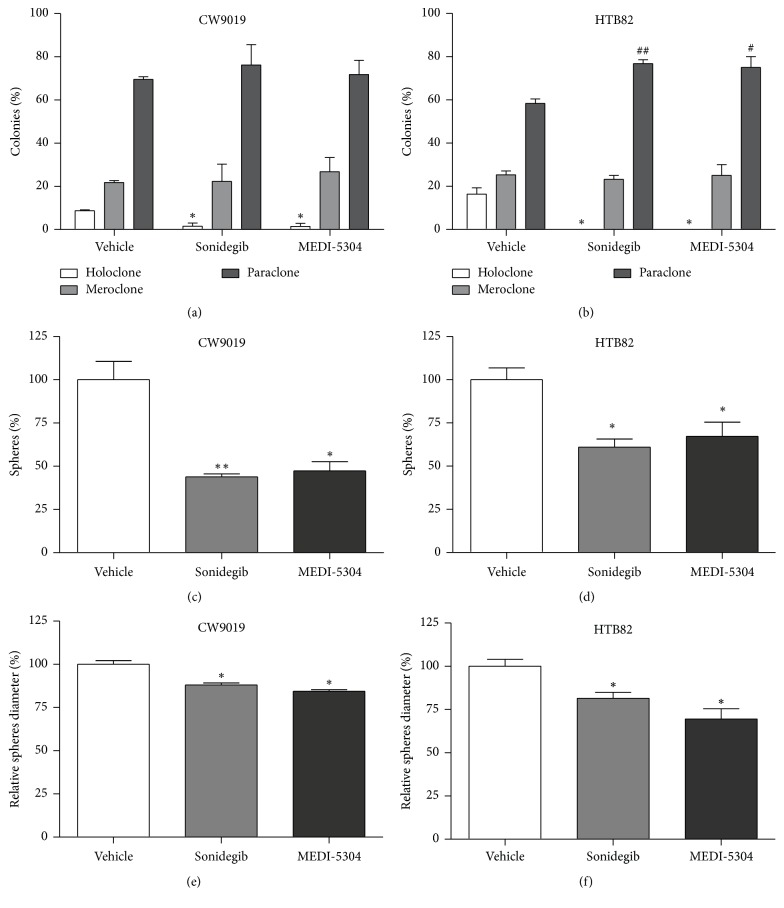
*HH* pathway inhibitors hindered holoclone and sphere formation. CW9019 and HTB82 cells were pretreated with Sonidegib (*SMO* inhibitor) and MEDI-5304 (HH ligand blocking antibody) 48 h prior to holoclone- and sphere-formation assays. ((a) and (b)) Plots representing the percentage of each colony type formed after* HH* pathway inhibitor treatment. Percentages of number (c and d) and diameter (e and f) of spheres formed after* HH* pathway inhibitor treatment. Values were referred to control cells (treated with vehicle) and expressed as mean ± SEM of three independent experiments. Significance: ^*∗*^*p* < 0.05; ^*∗∗*^*p* < 0.01; referred to holoclones of control cells and ^#^*p* < 0.05; ^##^*p* < 0.01 referred to paraclones of control cells.

The genetic downregulation of* IHH, DHH*, and* GLI1* by shRNA blocked the formation of holoclones in both cell lines, whereas SHH silencing produced no effects (Figures 4(a) and 4(b)). The total number of colonies was not affected in any condition (Supplementary Figure S1, A and B, in Supplementary Material available online at https://doi.org/10.1155/2017/7507380). Conversely, CW9010 and HTB82 shRNA-expressing cells maintained the same ability to form spheres as control cells (transfected with empty vector) (Supplementary Figure 1, C and D). However, cells transduced with* IHH, DHH* and* GLI1* shRNA vectors showed a marked reduction in sphere diameter (Figures 4(c) and 4(d)). All shRNAs showed efficient reduction in their respective targets (Figures 4(e)–4(h)). Pharmacologic* HH* inhibition rendered similar results. Thus, RMS cell lines pretreated with Sonidegib (*SMO* inhibitor) or MEDI-5304 (*SHH* and* IHH* blocking antibody) showed a significant reduction in holoclone number and a concomitant increase in the number of the most differentiated clones—meroclones and paraclones—only significant in the HTB82 cell line (Figures 5(a) and 5(b)), without affecting the total number of clones (Supplementary Figure 2, A and B); after the initial 2-day pretreatment, the clone formation assays were performed in the absence of drug to prevent possible interferences with cell proliferation during the growth of clones. Likewise, marked reductions in the number of spheres (Figures 5(c) and 5(d)) as well as in their diameter (Figures 5(e) and 5(f)) were observed after* HH* pathway inhibition in both cell lines. No significant decrease in cell viability was observed after either Sonidegib or MEDI-5304 pretreatment (Supplementary Figure 2, C and D).

### 3.5. Expression of Stem Cell Markers in Holoclones

**Figure 6:**
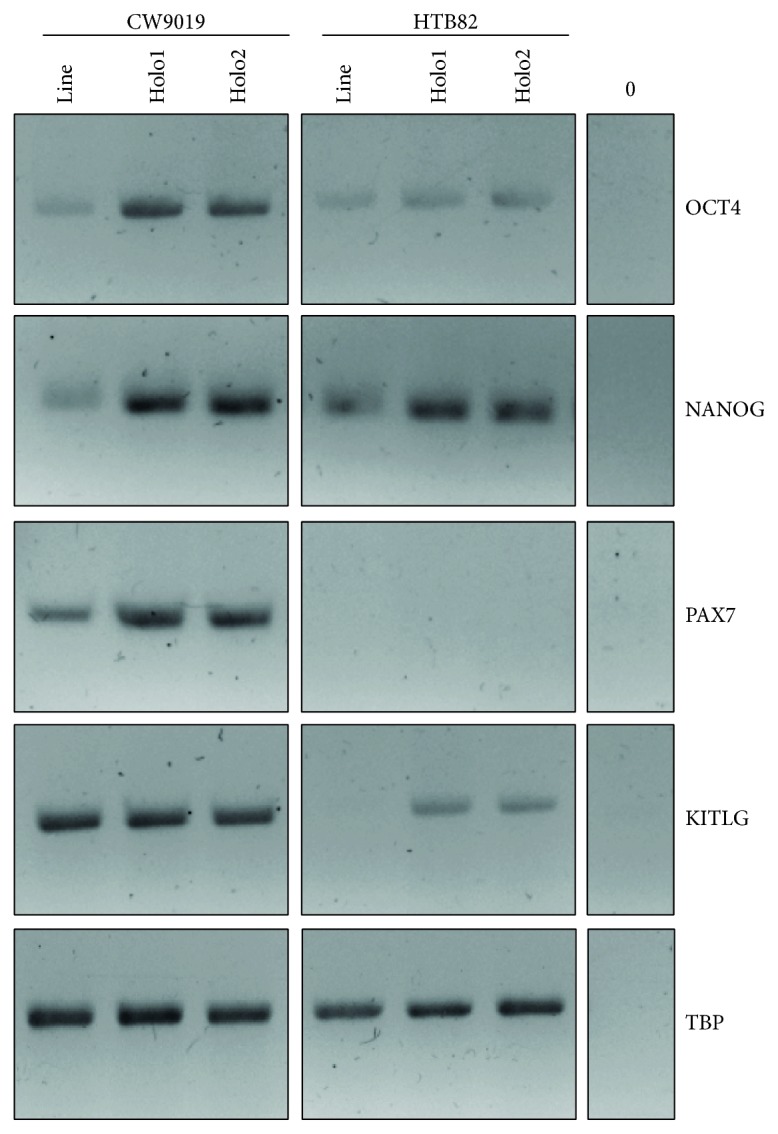
Holoclones showed induction of stem cell markers. mRNA levels of OCT4, NANOG, PAX7, and KITLG were evaluated in CW9019 and HTB82 cell lines. The expression levels of these markers were evaluated by conventional RT-PCR in total cell lines (line) and holoclones of first (holo1) and second (holo2) passages.

The expression levels of several stem cell markers were evaluated in CW9019 and HTB82 cell lines by PCR. Holoclones of both cell lines showed higher levels of OCT4 and NANOG compared to total cell lines. PAX7 was also increased in CW9019 (no expression of this marker was detected in HTB82). Conversely, KITLG was found to be upregulated in HTB82 holoclones, but no differences were observed in CW9019 for this marker (Figure 6). Other stem cell markers assayed (such as SOX2 and CD133) showed no variations (data not shown).

## 4. Discussion

Although the existence of cancer stem cells has been demonstrated and a growing body of evidence renders it obvious in some neoplasia, the case of RMS remains a challenge. Particularly in RMS, the existence of this rare subpopulation has only been reported for embryonal rhabdomyosarcoma and no evidence has been provided of its existence in the alveolar subtype, the most aggressive and prone-to-metastasizing form of RMS. Thus, three markers of CSC have been proposed in eRMS to date:* FGFR3, CD133*, and* SOX2* [12, 13, 15].* FGFR3*, the first CSC marker to be described in RMS, has been ruled out by subsequent studies [12]. Conversely, CD133+ cells have been successfully used to define a subpopulation with CSC properties in eRMS. However, a major drawback in the RMS field is the lack of useful CSC markers for the alveolar subtype, which has hampered the demonstration of the existence of this subpopulation in the most aggressive RMS subtype. Therefore, the use of other approaches, such as the formation of holoclones and spheres, may be an interesting alternative to study this subpopulation of cells in aRMS. Furthermore, the induction (compared to total cell lines) of stem cell markers (especially OCT4 and NANOG) suggests an enrichment of stem-like cells in the holoclones. The fact that an increase in stem cell markers was not observed in subsequent passages of clones suggests a maintenance mechanism of the proportions of putative stem cells in the clones.

The capability of a specific cell subpopulation to form holoclones has been reported in some cancers, including prostate [22], pancreatic [24], breast [41], non-small cell lung [42], and colorectal cancers [43]. The capability of cells to form spheres in culture has also been widely reported in cancer, even for the case of eRMS [12]. Both assays—based on the formation of supracellular structures—permit the enrichment of cells belonging to the stem cell subpopulation and therefore constitute a method for studying their peculiarities. The results presented herein demonstrate for the first time the capacity of RMS cells to form holoclones, and also confirm their capability to form spheres. Remarkably, only a minor percentage of cells (9–16%) was able to form holoclones, and only 0.5 to 2.5% of cells were able to form spheres. These low percentages support the hypothesis of the existence of a particular subpopulation of cells which would fit with the cancer stem cell theory. Interestingly, paraclones showed very low capacity to grow, and never formed colonies with more than 40–50 cells. This observation strongly suggests a reduced stemness of the cells contained in this type of clone. However, a discrepancy was found between the percentages of both supracellular structures. The lower efficiency of the sphere formation compared to holoclone formation may be explained in terms of higher stringency of the neurobasal media used for sphere cultures [44]. The fact that the holoclone-formation assay was performed in the habitual media of cells may explain the higher rates obtained, given that cells tend to accommodate faster and better in their habitual media. Moreover, the exclusive capability of cells from holoclones to form new holoclones is also suggestive of self-renewal of this fraction of cells. In this respect, the absolute lack of holoclones in cultures generated from meroclones or paraclones is particularly noteworthy. Therefore, the only cells able to originate the three types of colonies were those contained in holoclones. Furthermore, holoclone fraction was enriched in cultures derived from purified holoclones. In a similar fashion, the number of spheres obtained was clearly increased in subsequent passages from first-round cultured spheres.

The* HH* pathway has been previously shown to be crucial for CSC maintenance in several cancers [45–50]. The results herein presented showed a very clear activation of the* HH* pathway in the RMS stem cell-enriched subpopulation (holoclones and spheres). Thus, the main* HH* downstream target, the transcription factor* GLI1*, showed striking induction of its mRNA levels both in holoclones and spheres, thereby supporting a leading role of this pathway in stemness maintenance.* GLI1* is known to play a prooncogenic role in several cancers but is also characterized by its role in cancer stem cell maintenance [45, 49]. Furthermore,* GLI1* is able to activate the expression of two transcription factors,* NANOG* [51] and* SOX2* [49], known to be crucial for stemness maintenance. Beyond its possible role in cell stemness maintenance,* HH* signaling has been shown to be mutated or deregulated in many cancers in which it may support cell proliferation, tumor progression, metastasis and therapeutic resistance [52]. The relationship between* HH* signaling and RMS was first described by Hahn et al. in 1996 [30] and was further characterized in mice heterozygous for* PTCH1* which not only develop features consistent with Gorlin's syndrome, but also have a high incidence of eRMS [32]. A consistent activation of the pathway is well established and generally accepted in RMS [53]. Recently, Satheesha et al. reported impaired tumor formation under* HH* inhibitor treatment in eRMS [39]. However, the present report is the first to describe a possible pharmacologic application of* HH* inhibitors in reducing the number of stem-like cells in a PAX7/FOXO1-translocated aRMS cell line, thereby suggesting that these results could be extrapolated to the whole aRMS phenotype. Our results strongly suggest that a therapy based on* HH* pathway inhibition not only will act on cell proliferation but also may be able to reduce the capability of cells to generate new tumors (i.e., local relapses or metastasis). Involvement of the* HH* pathway in the promotion of cell invasiveness, metastasis, and tumor progression—albeit not in RMS—has previously been demonstrated in several neoplasia [54–58]. If we take into account the fact that the main cause of death from sarcoma—as in the majority of cancers—is the development and progression of metastasis, a possible therapy which targets both cell proliferation and tumor-initiating capacity may be a useful tool for impeding cancer progression, thereby improving patient survival. Therefore, the results herein presented reinforce the application of* HH* pathway inhibitors in RMS treatment.

On the other hand, a strong induction of* HH* ligand expression in spheres was clearly manifested. In this respect, evidence has accumulated that aberrant* HH* secretion by tumor cells may stimulate stromal cells near the tumor in a paracrine manner, including stimulation of tumor angiogenesis, extracellular matrix modification and secretion of* VEGF,* among others [59, 60]. We may speculate that, as spheres grow, cells located in the central part become isolated, and nutritive deprivation and/or hypoxia may activate this previously known mechanism of triggering angiogenesis. Given that holoclones grow in a monolayer, they may not suffer this nutritional deprivation and therefore may not activate this mechanism. However, this is mere speculation and further experiments will be required to support this hypothesis.

Regarding the possible clinical application of the results herein presented, the fact that* HH* pathway pharmacologic inhibition reduces the formation of both holoclones and spheres is particularly noteworthy. Thus, both pharmacologic approaches (*SMO* inhibitor Sonidegib and* HH*-blocking antibody MEDI-5304) showed significant reductions in the formation of spheres and holoclones. However, although the genetic inhibition of* IHH, DHH* and* GLI1 *hampered the formation of holoclones, it was able only to reduce sphere diameter (no decrease in sphere number was observed). This fact suggests that these Hedgehog components may be more crucial and specific for the initiation of holoclones than spheres. In any event, these results support the* HH* pathway as a key therapeutic target against RMS stem-like cells. Thus, our results suggest, for the first time, the possible use of* HH* ligand-specific inhibitors to block RMS stemness. In this respect, antagonists of* SMO* were entered in phase I and II clinical trials, with encouraging results in* HH*-driven neoplasia, particularly in medulloblastoma and basal cell carcinoma (BCC) [61, 62]. More recently, the* SMO* inhibitor Vismodegib became the first* HH* signaling pathway-targeting agent to be approved by the US Food and Drug Administration (FDA) for the treatment of metastatic or locally advanced BCC [63]. Given that the* HH* pathway is strongly activated in cancer stem cells and inhibition of the pathway clearly reduces CSC, we can affirm that, besides the inhibition of tumor growth (which is the commonest criterion for selecting the applicability of a given drug), the use of* HH* inhibitors may help to lower the number of cancer stem cells, at least in RMS. Targeting of this particularly malignant—albeit rare—subpopulation may have great potential in preventing local relapses and metastases, thus improving survival.

## Supplementary Material

Supplementary figure 1. The genetic downregulation of HH ligands and GLI1 reduced neither clonogenicity nor sphere formation. Supplementary figure 2. Pretreatment with Sonidegib and MEDI-5304 reduced neither clonogenicity nor cell viability.
