# Supplementary material for: Hedgehog Pathway Inhibition Hampers Sphere and Holoclone Formation in Rhabdomyosarcoma
Source: Stem Cells Int. 2017 Jan 24;2017:7507380. doi: 10.1155/2017/7507380 (PMC5294584; doi:10.1155/2017/7507380)
Supplement: Supplementary file 1 — Supplementary figure 1. The genetic downregulation of HH ligands and GLI1 reduced neither clonogenicity nor sphere formation. Supplementary figure 2. Pretreatment with Sonidegib and MEDI-5304 reduced neither clonogenicity nor cell viability. [file 7507380.f1.pdf]

Supplementary Figure legends

Supplementary figure 1. The genetic downregulation of *HH* ligands and *GLI1* reduced neither clonogenicity nor sphere formation. Percentage of total colonies (**A** and **B**) and spheres (**C** and **D**) formed by *SHH, IHH, DHH* and *GLI1* shRNA-expressing cells (CW9019 and HTB82). Values were referred to control cells (transfected with pGIPZ empty vector) and expressed as mean ± SEM of three independent experiments.

Supplementary figure 2. Pretreatment with Sonidegib and MEDI-5304 reduced neither clonogenicity nor cell viability. Percentage of total colonies (**A** and **B**) and cell viability (**C** and **D**) after HH pathway inhibitor treatment in CW9019 and HTB82 cell lines, respectively. Values were referred to control cells (treated with vehicle) and expressed as mean ± SEM of three independent experiments.

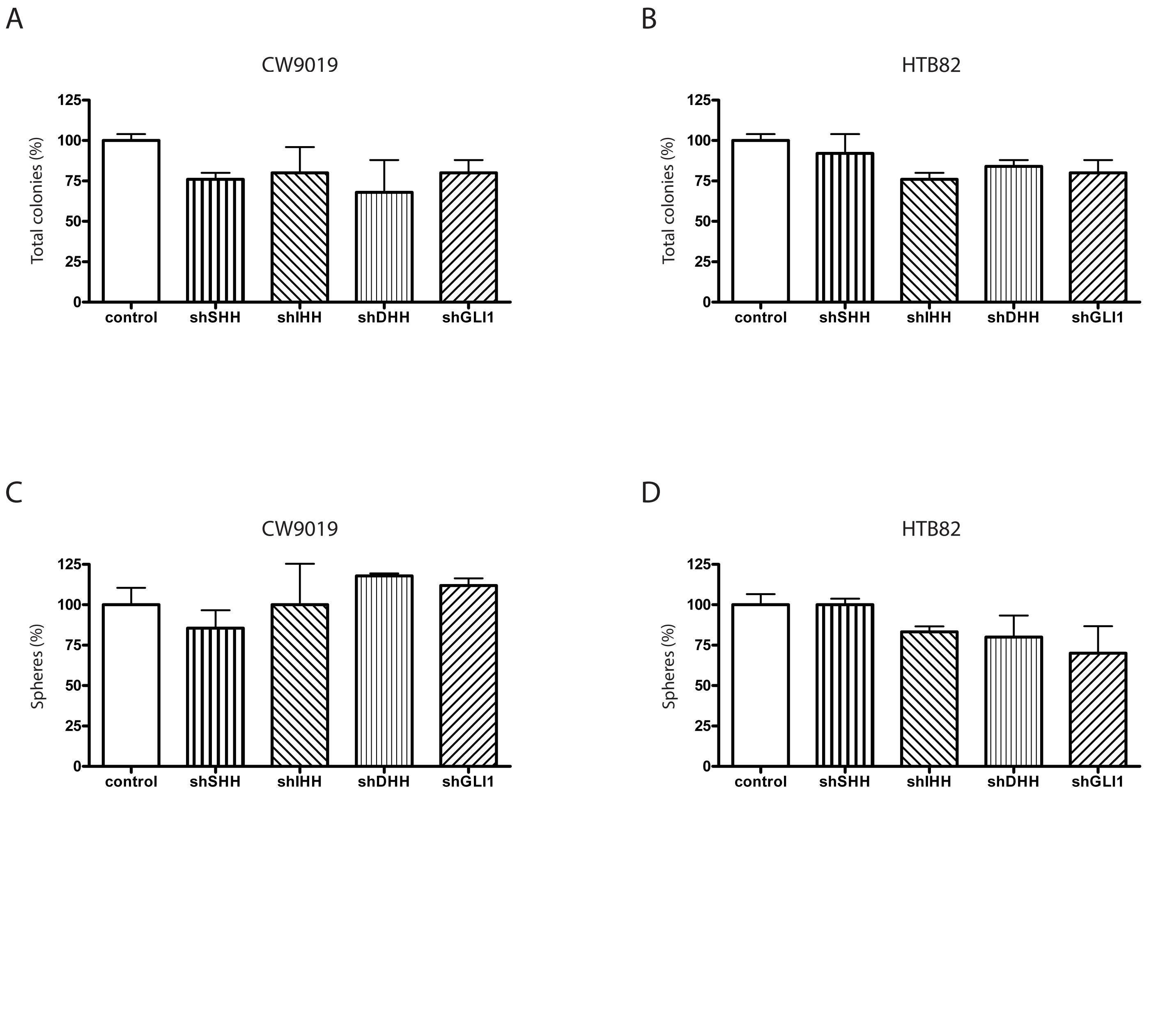
A
CW9019
Total colonies (%)
125
100
75
50
25
0
control
shSHH
shIHH
shDHH
shGLI1
B
HTB82
Total colonies (%)
C
CW9019
Spheres (%)
D
HTB82
Spheres (%)

Figure S1

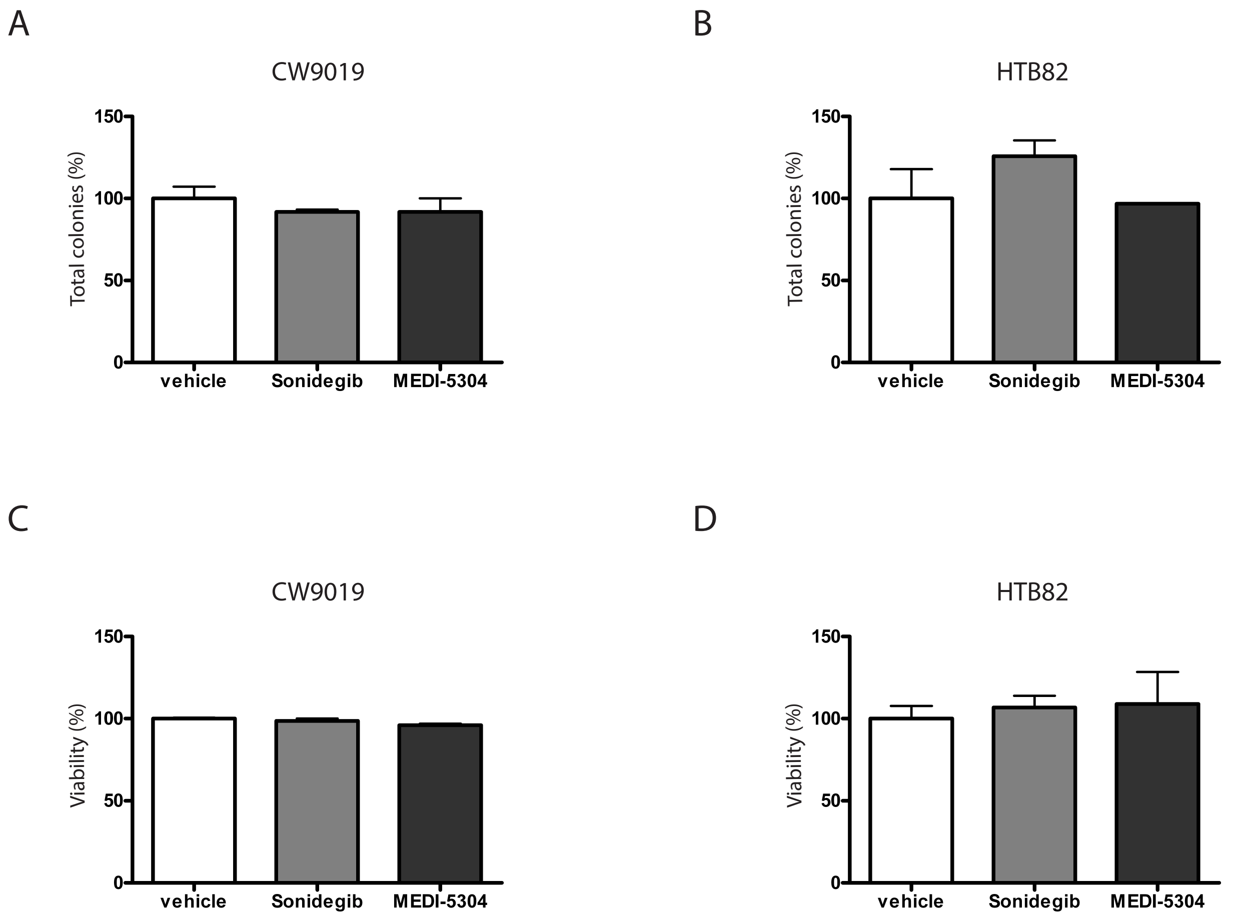
A
CW9019
Total colonies (%)
150
100
50
0
vehicle
Sonidegib
MEDI-5304
B
HTB82
Total colonies (%)
150
100
50
0
vehicle
Sonidegib
MEDI-5304
C
CW9019
Viability (%)
150
100
50
0
vehicle
Sonidegib
MEDI-5304
D
HTB82
Viability (%)
150
100
50
0
vehicle
Sonidegib
MEDI-5304

Figure S2
